# The Medication-Related Burden Quality of Life (MRB-QoL) tool: A confirmatory factor analysis of the Arabic version

**DOI:** 10.1016/j.rcsop.2025.100568

**Published:** 2025-01-15

**Authors:** Sundos Q. Al-Ebrahim, Khadija Hafidh, Mahir Jallo, Mais M. Mauwfak, Mohamed Nassef, Hamzah Alzubaidi, Jeff Harrison, Timothy F. Chen, Mohammed A. Mohammed

**Affiliations:** a School of Pharmacy, Faculty of Medical and Health Sciences, The University of Auckland, Auckland, New Zealand; b Internal Medicine Department, Rashid Hospital, Dubai Academic Health Corporation, Dubai, United Arab Emirates; c School of Medicine, Mohammed Bin Rashid College of Medicine and Health Sciences, Dubai, United Arab Emirates; d Internal Medicine Department, Gulf Medical University, Ajman, United Arab Emirates; e Internal Medicine Department, Thumbay University Hospital, Ajman, United Arab Emirates; f Anesthesia and Critical Care Medicine Department, Al Qassimi Hospital, Emirates Health Services, Sharjah, United Arab Emirates; g College of Pharmacy, University of Sharjah, Sharjah, United Arab Emirates; h School of Pharmacy, Faculty of Medicine and Health, The University of Sydney, Sydney, Australia

**Keywords:** Arabic, MRB-QoL, Confirmatory factor analysis, Psychometric testing, Validity, Reliability, Conceptual model, Factor structure

## Abstract

**Background:**

The Medication-Related Burden Quality of Life (MRB-QoL) Arabic version is a 31-item valid and reliable measure of medication burden on functioning and well-being.

**Objective:**

To examine the factor structure of the MRB-QoL Arabic in a sample of adults living with long-term conditions (LTC).

**Methods:**

Three hundred forty-three patients (≥ 18 years old) living with at least one LTC were recruited from 4 tertiary hospitals in the United Arab Emirates. Confirmatory factor analysis (CFA) was performed using Maximum likelihood estimation with bootstrap. Two models (first order and second order) were examined. Model fit indices, composite reliability (CR), and average variance extracted (AVE) were used to assess the model's goodness of fit, reliability, and convergent/discriminant validity, respectively. The model's fit was evaluated using absolute fit, comparative fit, and parsimony-adjusted indices. The RMSEA and SRMR ≤0.08, χ^2^/df < 5, and CFI, IFI, and TLI ≥ 0.90 were considered indicators of good model fit. PNFI and PCFI >0.5 were also considered as indicators of good fit. CR ≥ 0.7, AVE ≥ 0.5, and AVE greater than squared factors correlation were considered as evidence indicating reliability, convergent validity, and discriminant validity, respectively.

**Results:**

The first-order model showed an excellent fit (χ2/df = 3.262, RMSEA = 0.08, SRMR = 0.05, CFI = 0.913, TLI = 0.914, IFI = 0.914, PNFI = 0.810, PCFI = 0.841) as did the second-order model (χ2/df = 2.845, RMSEA = 0.073, SRMR = 0.072, CFI = 0.934, TLI = 0.923, IFI = 0.915, PNFI = 0.820, PCFI = 0.851). All domains of the MRB-QoL met the convergent/discriminant validity and reliability criteria.

**Conclusions:**

The study supports the factor structure from previous research and confirms the MRB-QoL Arabic as a valid and reliable measure. This tool can be used to assess medicines burden from patient perspectives and facilitate person-centred care in medicines optimisation services across Arabic-speaking countries.

## 1 Introduction

Medications are commonly used healthcare interventions for the prevention and treatment of various medical conditions; however, they come with an inherent burden when used inappropriately and for a prolonged duration. For many patients with long-term conditions (LTC), managing medication routines requires substantial time, resources, and commitment, which can be burdensome for some individuals.^1^ This burden is often framed as a medication-related burden (MRB),^2^ referring to patients' workload in managing their medications and the impact of this on daily functioning and overall well-being.^3^, ^4^, ^5^, ^6^ Some individuals with LTC are more susceptible to MRB,^4^ which can significantly impact an individual's social, psychological, and physical well-being.^2^^,^^3^^,^^7^^,^^8^ Understanding and quantifying the impacts of MRB on patient well-being requires using valid and reliable patient-reported measures. Although most quality of life measures commonly used in medication optimisation interventions cover physical, social, and psychological domains,^9^^,^^10^ they are not suitable for assessing the burden of medication on health and well-being.^2^^,^^9^^,^^11^ Over the past decade, there has been growing interest in developing treatment-specific measures more suitable for assessing humanistic outcomes in medication optimisation interventions.^7^^,^^12^, ^13^, ^14^, ^15^, ^16^, ^17^, ^18^, ^19^, ^20^ One such measure includes the Medication-Related Burden Quality of Life (MRB-QoL) tool.^20^

The MRB-QoL tool is a patient-reported measure of medication burden on functioning and well-being developed in Australia^20^ and was recently translated and culturally adapted into Arabic.^21^ The development process of the original MRB-QoL involved 3 phases, and was informed by the integration of 3 fundamental concepts, including pharmaceutical care, health-related quality of life, and MRB. By combining these concepts, the tool provides a holistic approach to understanding the impact of medications on patients' lives, emphasising not only the physical aspects of health but also broader dimensions of well-being. The Arabic version was found to be culturally appropriate, valid, and reliable for measuring MRB in Arabic-speaking individuals with LTC.^21^^,^^22^ Standard written Arabic (Fus-ha) was used in the MRB-QoL Arabic version to make it understandable and applicable across Arabic-speaking countries. Despite the proven validity and reliability of the Arabic MRB-QoL measure,^21^^,^^22^ its factor structure has not been evaluated. This study aimed to extend the work on the culturally adapted and psychometrically tested Arabic version by evaluating its factor structure through confirmatory factor analysis (CFA). From this, we aim to gain further insights into its underlying factor structure, validity, and reliability.

## 2 Methods

### 2.1 Study design

A multicentre exploratory cross-sectional survey was conducted from November 2023 to August 2024. In this study, we followed the consensus-based standards for selecting health measurement instruments (COSMIN) taxonomy.^23^ This was to avoid the potential confusion caused by the variations in terminologies used to describe measurement properties and their definitions. To ensure that all standards were considered during the study's design, implementation, and reporting, we used the COSMIN study design checklist^24^ and reporting guidelines for studies on measurement properties.^25^

### 2.2 Participants and setting

Participants who were 18 years and older, living with at least one LTC, taking at least one long-term medication, and able to complete the survey in Arabic were included in the study. Those with cognitive impairment and individuals with terminal illness, vision, or hearing difficulties were excluded.

A minimum of 3 to 10 participants per item is recommended for factor analyses.^26^^,^^27^ This study estimated the sample size using a 10:1 participant-to-item ratio (i.e. 10 × 31 = 310). To account for potential missing data, a 10 % increase was considered, making the total sample size 343 participants. A consecutive sampling cluster approach was implemented in 4 tertiary hospitals across 3 cities in the UAE.

### 2.3 The Arabic MRB-QoL measure

Al-Ebrahim SQ et al. (2024)^21^ recently translated and culturally adapted the MRB-QoL into Arabic. The researchers used a rigorous content validation method, which included the e-modified Delphi with an expert panel and cognitive debriefings with end users. The Arabic MRB-QoL tool was also tested for validity and reliability^22^ and found to have good construct validity, including structural, known-group, convergent, and discriminant validity. It also showed excellent reliability, including high internal consistency, low measurement error, and good test-retest reliability. The MRB-QoL Arabic version retains the original 31 items but categorises them into 4 domains: routine and regimen complexity (RRC, 11 items), psychosocial burden (PsySB, 9 items), functional and role limitation (FRL, 8 items), and therapeutic relationship (TR, 3 items). In contrast, the original English categorise the same 31 items into 5 domains: “Routine and Regimen Complexity” (RRC, 11 items), “Psychological Burden” (PsyB, 6 items), “Functional and Role Limitation” (FRL, 7 items), “Therapeutic Relationship” (TR, 3 items), and “Social Burden” (SB, 4 items). The psychometric testing of the original MRB-QoL on community-dwelling adults in Australia demonstrated good evidence for validity and internal consistency reliability.^20^ In addition to Arabic, the MRB-QoL was also translated and culturally adapted into German.^28^

### 2.4 Data collection

Participants were approached during their visit to the study site, screened for eligibility, and informed about the study. Participants who agreed to participate in the study received copies of the participant information sheet, consent form, and the MRB-QoL Arabic. Socio-demographic and clinical data were extracted from participants' medical records.

### 2.5 Data analysis

CFA was conducted to validate the factor structure of the Arabic MRB-QoL identified by the exploratory factor analysis (EFA).^22^ This analysis allowed us to examine the dimensionality of latent constructs, confirm if the observed variables (i.e. questionnaire items or indicators) corresponded to a specific construct or dimension, and evaluate the significance of the proposed measurement model.^29^ This analysis is a more rigorous and systematic test of factor structures than EFA. CFA involves 5 key steps: model specification, identification, estimation, assessment, and respecification.^29^

Each item was considered to have a latent construct and a measurement error, represented by unidirectional arrows. Correlations between variables within the model were represented using bidirectional arrows. Before performing CFA, the measurement scales of the items were examined for skewness and kurtosis. Maximum likelihood estimation with bootstrap was used to address issues related to the distribution of the data (i.e. non-normality) and the constraints of a moderate sample size in the study. Measurement errors were calculated as (1-reliability) times the variance.^30^ Upon examination of the dependent variable and the resultant scales' items, multivariate non-normality was detected, as reflected by Mardia's coefficient (255.91, critical ratio = 52.39). Consequently, maximum likelihood estimation with bootstrapping was employed to handle non-normally distributed data. Continuous variables were summarised using medians and interquartile ranges, while categorical variables were reported using frequencies and percentages.

**Fig. 1:**
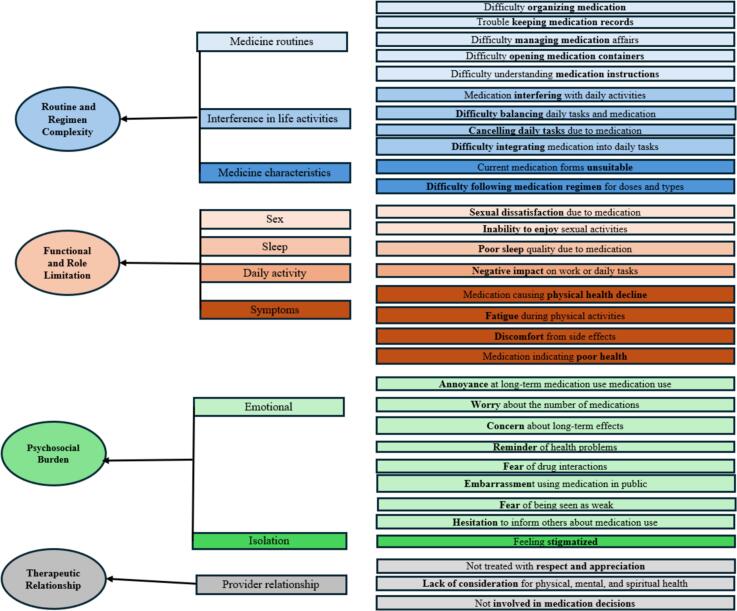
Conceptual framework of the Arabic MRB-Qol measure.

Two measurement models, the first-order and second-order models, were examined. In the first-order model, we tested the fit of the model in the 31 items MRB-QoL grouped into 4 correlated factors (domains), accounting for all common variance among the items. In the second-order model, the model fit was tested in the proposed second-order structure, where the 4 first-order factors were aggregated into one latent variable (i.e. total MRB-QoL). This model is a hierarchical model where items load onto the 4 first-order factors, and the factors are aggregated into a second-order latent variable represented as the overall MRB-QoL. The proposed factor structure was informed by the original MRB-QoL conceptual framework^20^ and the factor solution identified in the EFA of the MRB-QoL Arabic version (Fig. 1). The second-order model was examined for 2 reasons: first, the EFA used in the development of MRB-QoL Arabic does not identify second-order constructs or provide statistical evidence for their existence; and secondly, providing further evidence on factor structure is needed for researchers who may be interested in using the total MRB-QoL score rather than domain level scores.

The goodness of fit in each model was evaluated using the following indices: absolute fit, comparative fit, and parsimony-adjusted indices.^31^^,^^32^ Absolute fit indices include the Chi-square test (χ^2^), Chi-square divided by degrees of freedom (χ^2^/df), Root Mean Square Error of Approximation (RMSEA), and Standardised Root Mean Square Residual (SRMR). Comparative fit indices include the Comparative Fit Index (CFI), Tucker Lewis Index (TLI), and Incremental Fit Index (IFI). Parsimony-adjusted indices include the Parsimony Normed Fit Index (PNFI) and the Parsimony Comparative Fit Index (PCFI).^32^, ^33^, ^34^, ^35^ The model fit was determined using the following criteria: RMSEA ≤0.08, a non-significant χ^2^, χ^2^/df < 5, SRMR ≤0.08 ^36, 37^ as well as CFI, IFI, and TLI ≥ 0.90 with PCFI and PNFI values >0.5.^36^ The factor loadings were examined to determine the contribution of items and domains to the models, with loading ≥0.5 considered acceptable.^36^^,^^38^^,^^39^ In this study, we reported the 90 % confidence interval (CI) for RMSEA, following standard practice in structural equation modelling to enhance sensitivity to model fit^40^^,^^41^and ensure consistency with similar treatment burden measures.^42^^,^^43^ Standardised factor loadings below 0.5 were flagged for further examination to guide decisions on item removal or retention based on their impact on model fit. The Akaike Information Criterion (AIC) and Bayesian Information Criterion (BIC) were used to compare the fit of the 2 measurement models, with lower values indicating a better fit.^36^^,^^37^

Composite Reliability (CR) measures how well the variables represent a construct in structural equation modelling.^44^ A latent construct must achieve a CR of at least 0.7 to ensure construct reliability,^45^ indicating that all items consistently measure their respective constructs.

Convergent validity was assessed by calculating each construct's Average Variance Extracted (AVE), with AVE ≥ 0.5 indicating convergent validity.^46^ In addition, standardised factor loadings should exceed 0.50 and be statistically significant to confirm strong convergence on a common factor. Discriminant validity was evaluated using variance-extracted tests according to the Fornell-Larcker criterion,^47^ with the AVE value exceeding the squared coefficient with other constructs indicating evidence for discriminant validity.^47^, ^48^, ^49^, ^50^ Additionally, a correlation between factor pairs that was not excessively high (> 0.85) indicated evidence of discriminant validity.^51^

CFA was performed using IBM SPSS Amos 29.0.0 (IBM, New York, USA) and SPSS version 29 (SPSS Inc., Chicago, USA). All reported *p*-values were based on two-sided tests and were compared with a significance level of 5 %.

### 2.6 Ethics approval

Ethics approval was obtained from the Auckland Health Research Ethics Committee at the University of Auckland, New Zealand (Approval No. AH24337), the Research Ethics Committee of the UAE Ministry of Health and Prevention (Approval No. MOHAP/DXB-REC/O.N·D/No.101/2022), and the Dubai Scientific Research Ethics Committee at the Dubai Health Authority, UAE (Approval No. DSREC-01/2023_13).

## 3 Results

### 3.1 Participant characteristics

Three hundred forty-three patients completed the MRB-QoL survey across 4 tertiary hospitals in 3 cities in the UAE. Eighty-six patients were recruited from three hospitals, while 85 patients were recruited from the fourth hospital. The median (IQR) age was 45 (36–54) years, and 49.9 % of the study participants were females. Over 39 % of participants were UAE citizens, and about half (54.5 %) were unemployed. The median (IQR) number of prescription medicines and medical conditions were 3 (1–4) and 4 (3–5), respectively. Nearly half of the sample (*n* = 171) were on polypharmacy (≥ 5 medicines) and 7.9 % (*n* = 27) had hyper-polypharmacy (≥ 10 medicines).^52^ About 80 % of the study participants had 3 or more LTC (Table 1).

**Table 1:** Characteristics of the study participants (*N* = 343).

Participant characteristic	
Age in years (IQR)	45 (36–54)
Male gender, n (%)	172 (50.1 %)
Ethnicity	
Arab (UAE), n (%)	134 (39.1 %)
Arab (non-UAE), n (%)	199 (58 %)
Asian, n (%)	6 (1.7 %)
Others, n (%)	4 (1.2 %)
Occupation	184 (54.5 %)
Unemployed, n (%)	
Employed, n (%)	145 (42.3 %)
Retired, n (%)	10 (2.9 %)
Student, n (%)	4 (1.2 %)
Presence of co-morbidities, n (%)	343 (100 %)
< 3 medical conditions, n (%)	71 (20.7 %)
≥ 3 medical conditions, n (%)	272 (79.3 %)
Number of medical conditions (IQR)	4 (3–5)
Number of prescription medicines (IQR)	3 (1–4)
Number of over-the-counter medicines (IQR)	2 (1–3)

IQR: Interquartile range.

### 3.2 Confirmatory factor analysis (CFA)

No items were missing, and all the 343 responses were analysed. The correlation matrix analysis showed that no correlations exceeded 0.85, indicating that multicollinearity was not a concern.^40^

### 3.3 First-order CFA

**Fig. 2:**
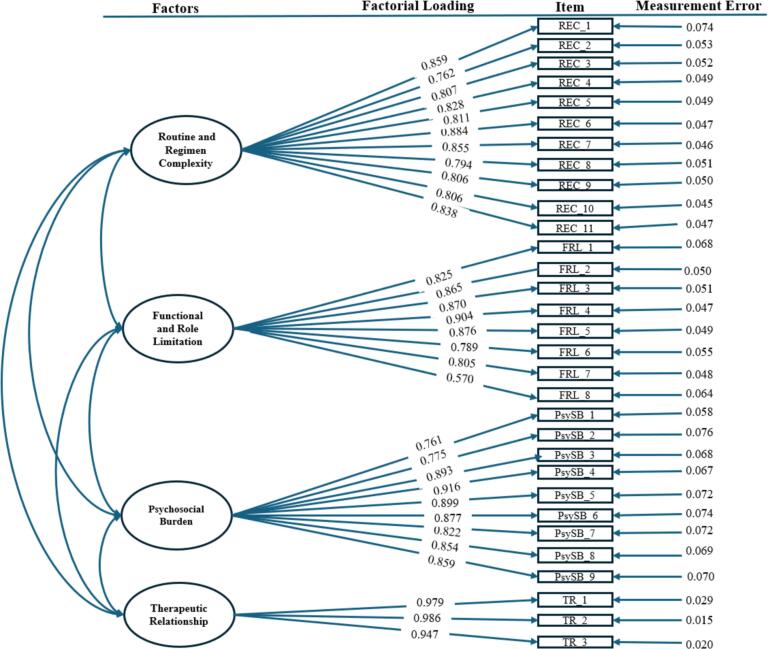
Final first-factor model of the Arabic MRB-Qol measure with standardised parameter estimates.

The first-order model showed a good fit. The χ2 value for the model fit was significant (χ2 (428) = 1395.928, *P* ≤ 0.05), indicating a misfit between the data and the model. A more precise metric (i.e. χ2/df)^53^ was assessed, which showed satisfactory model fit (χ2/df = 3.26, which is <5). All other fit indices also indicated an excellent model: CFI = 0.913, TLI = 0.914, NFI = 0.987, IFI = 0.914, PNFI = 0.810, PCFI = 0.841, RMSEA = 0.08 [90 % CI: 0.076–0.085], and SRMR = 0.05 (Table 2). CFA confirmed inter-correlations among factors underlying the MRB-QoL (Table 3). The item loading values ranged from 0.57 to 0.97, with a mean of 0.83, all exceeding the acceptable threshold of 0.50 (Fig. 2).

**Table 2:** Confirmatory factor analysis (CFA) first-order and second-order models, and goodness-of-fit indices.

Fit indices	Recommended value	First order model	Second order model
*P* value	> 0.05	0.000	0.000
**χ**2/df	< 5	3.262	2.845
SRMR	<0.08	0.05	0.072
RMSEA	≤ 0.08	0.080	0.073
TLI	≥ 0.9	0.914	0.923
CFI	≥ 0.9	0.913	0.934
IFI	≥ 0.9	0.914	0.915
PNFI	> 0.5	0.810	0.820
PCFI	> 0.5	0.841	0.851
AIC	–	1531.928	1331.514
BIC	–	1792.894	1692.261

χ^2^/df: Chi-square/degrees of freedom, SRMR: Standardised Root Mean Square Residual, RMSEA: Root Mean Square Error of Approximation, TLI: Tucker Lewis Index, CFI: Comparative Fit Index, IFI: Incremental Fit Index, PNFI: Parsimony Normed Fit Index, PCFI: Parsimony Comparative Fit Index, AIC: Akaike Information Criterion, BIC: Bayesian Information Criterion.

**Table 3:** Composite reliability, the square root of the average variance extracted, and correlations between constructs.

Factors	CR	AVE	RRC	FRL	PsySB	TR
RRC	0.960	0.687	**0.829**⁎			
FRL	0.941	0.670	0.112⁎⁎	**0.819**⁎		
PsySB	0.960	0.726	0.118⁎⁎	0.080⁎⁎	**0.852**⁎	
TR	0.980	0.942	0.295⁎⁎	0.274⁎⁎	0.039⁎⁎	**0.971**⁎

RRC: Routine and regimen complexity, FRL: Functional and role limitation, PsySB: Psychosocial burden, TR: Therapeutic relationship, CR: Composite reliability, AVE: Average variance extracted.

⁎ The square root of the average variance extracted is shown in bold.

⁎⁎ The correlations between constructs are shown off-diagonal.

### 3.4 Second-order CFA

**Fig. 3:**
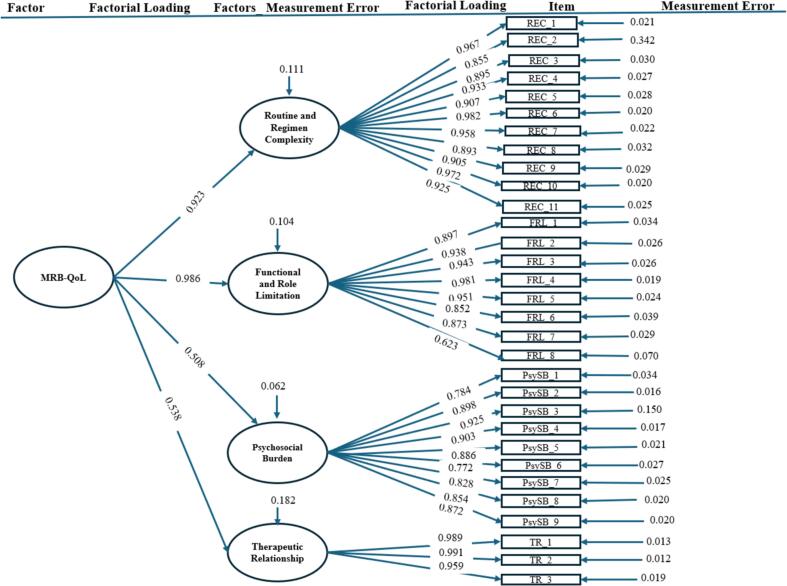
Final two-factor model of the Arabic MRB-QoL measure with standardised parameter estimates.

Although the χ2 value for the model signalled some misfit between the data and the model*,* the second-order model (Fig. 3, Table 2) also showed excellent fit across all fit indices (χ2 = 1143.514, *P* < 0.001, χ2/df = 2.845, RMSEA = 0.073 [90 % CI: 0.068–0.078], SRMR = 0.072, CFI = 0.934, TLI = 0.923, IFI = 0.915, PNFI = 0.820, PCFI = 0.851). In the second-order model, slightly higher loadings were observed compared to the first model, with minimal differences (≤ 0.18). This model also showed a slight improvement in overall fit, indicated by improved fit indices and lower AIC and BIC values (AIC = 1331.514, BIC = 1692.261) relative to the first-order model (AIC = 1531.928, BIC = 1792.894).

### 3.5 Validity and reliability

First-order CFA allows the assessment of the validity and reliability of individual items. Adding a second-order factor does not affect item validity and reliability estimates. Thus, the validity and reliability of the 31 items would not be affected by the type of model.^54^ Table 3 presents the latent construct of the proposed measurement model that showed strong discriminant and convergent validity, as well as high CR. All MRB-QoL domains met the discriminant validity criteria. Each domain's AVE exceeded the squared correlations with other domains, and all factor intercorrelations were below 0.85, meeting the Fornell-Larcker criterion.^47^ The AVE value for all MRB-QoL domains exceeded the 50 % threshold, and all item factor loadings were above 0.5, indicating convergent validity. All MRB-QoL domains also had the CR above the threshold (0.7): RRC (0.960), FRL (0.941), PsySB (0.960), and TR (0.980).

## 4 Discussion

There is growing international recognition of the importance of patient-reported measures in assessing the impact of MRB on functional and psychosocial well-being in individuals with LTC*.* The MRB-QoL Arabic version, recently developed and validated for use in Arabic-speaking countries, represents an important advancement in assessing MRB. Despite robust evidence on its measurement properties, the factor structure of MRB-QoL Arabic has not been empirically evaluated. This study is the first to apply CFA to investigate the factor structure of MRB-QoL Arabic using 2 measurement models (first-order and second-order) and to provide additional evidence supporting its validity and reliability. The underlying factor structures in the 2 models were informed by the conceptual framework used in the original MRB-QoL^20^ and the EFA results in the Arabic version.^22^ Absolute, comparative, and parsimony-adjusted fit indices were used to assess how well the model fits the data and explore the evidence for a higher-order construct. Our findings showed that the MRB-QoL Arabic tool is a reliable and valid measure for assessing MRB on the functioning and well-being of people with LTC. Both first-order and second-order models showed a good fit, confirming the conceptual framework of the MRB-QoL Arabic measure. All domains demonstrated high construct reliability and good convergent and discriminant validity.

Developing a standardised instrument, such as the Arabic MRB-QoL, involves 2 key stages^55^: (1) exploratory studies that create hypothesised measurement models from empirical data and (2) confirmatory studies that validate these models with new data from the target population. For the MRB-QoL, the exploratory stage has been extensively assessed in previous studies.^21^^,^^22^ However, further investigation is needed to confirm its factor structure and provide deeper insights into each MRB-QoL construct. This study completes an exploratory-confirmatory research cycle by validating the Arabic MRB-QoL tool through CFA while confirming its construct reliability and validity. Compared to EFA, CFA offers a more systematic and stringent evaluation of alternative factor structures.^56^ CFA involves defining and estimating factor structure models and identifying latent variables that explain covariances among observed variables.^29^ This requires identifying plausible factor patterns from existing theoretical or empirical research, which are then statistically evaluated using sample data. Both validity and reliability are conceptualised differently in CFA compared to exploratory analysis.^47^^,^^57^ In CFA, factor loadings are interpreted as regression coefficients of observed variables on latent variables.^29^ At the first-order level, factor loadings indicate the validity of observed variables relative to latent variables. Likewise, composite reliability may be more accurate for reliability assessment than Cronbach's alpha as it considers factor loadings and error variances, particularly in scales with multidimensional constructs.^47^ Thus, performing both exploratory and confirmatory analyses of the measurement properties of an instrument provides better insights into its validity, reliability, and other psychometric properties. Psychometric evaluations in most treatment burden measures have predominantly focused on EFA, with few extending to CFA.^42^^,^^43^^,^^58^, ^59^, ^60^ No existing measures of treatment burden in Arabic have incorporated data on CFA.^61^, ^62^, ^63^, ^64^ This highlights the need for healthcare providers and researchers to prioritise measures that have undergone comprehensive psychometric evaluations in clinical settings. Additionally, developing training programs to emphasise the importance of these evaluations can further enhance the robustness and applicability of such measures.

The findings of this study confirmed that the MRB-QoL tool is valid and reliable, highlighting its robustness as a patient-reported outcome measure for assessing MRB in practice and research. These results align with previously reported data on the EFA, validity, and reliability of both the Arabic version^22^ and the original tool,^20^ confirming its conceptual framework. This study provides a foundation for future research to expand the applicability of the Arabic MRB-QoL tool through methods such as differential item functioning analyses, measurement invariance testing, structural equation modelling, latent class analysis, and multigroup CFA.

In CFA, higher-order factor models use an additional matrix to illustrate the loadings of first-order factors onto higher-order factors, providing a more parsimonious explanation of their covariation.^54^ In this study, the second-order model showed excellent fit indices with lower AIC/BIC values, providing evidence for a robust higher-order factor that explains the covariation across the 4 domains of the Arabic MRB-QoL tool. These findings indicate that this tool can be used to assess individual domains of MRB and generate an overall burden score. This allows the tool to be used at both the domain level and as a single aggregate score, depending on the research or clinical context and setting. Although the second-order model offers a holistic view of medication burden and demonstrates superior fit indices, the first-order model is preferable when domain-specific insights are required, such as for research into individual domains or targeted clinical interventions. These findings align with the results of Lee MK et al. (2020),^43^ who used CFA to confirm the conceptual factor structure of a treatment burden measure and supported the existence of two second-order factors.

This study utilised data from hospital outpatients, in contrast to the original MRB-QoL instrument, which was developed using data from community-dwelling patients. Outpatients typically interact more frequently with healthcare professionals and participate more actively in healthcare than community-dwelling individuals.^65^ While this difference broadens the tool's applicability, MRB may vary between these populations. Future research should validate the MRB-QoL in community-dwelling populations to ensure its generalisability to diverse patient groups. Citizens of the UAE and other Arabic-speaking countries share strong linguistic and cultural similarities. The use of standard written Arabic (Fus-ha), the formal written language common across Arabic-speaking regions, enhances the tool's acceptability in diverse Arabic-speaking contexts. However, variations in healthcare systems, such as differences in medication access, insurance coverage, and medication management practices, could affect the tool's applicability. Future multinational validation studies should evaluate how these variations influence the tool's performance across diverse healthcare settings.

### 4.1 Implications for practice and research

The Arabic MRB-QoL tool can be a useful patient-reported outcome measure in clinical practice for identifying patient subgroups at increased risk of poor medication-related health outcomes. Furthermore, it holds promise for health outcome research in evaluating the impact of medicines optimisation services. Its comprehensive nature makes it suitable for diverse healthcare settings and patients. Healthcare providers can use the MRB-QoL to identify patients with high MRB and those struggling with adherence issues or feeling overwhelmed by medication routines and/or regimen complexity. The MRB-QoL provides insights into the patient's experience that cannot be obtained from objective clinical measures often documented on medical records. This tool allows patients to express their MRB in a more structured and quantified manner. This, in turn, facilitates patient-clinician communication for shared decision-making to optimise medicines use and patient health outcomes. Therefore, the clinical utility of the MRB-QoL should be evaluated in future research. Additionally, a large-scale prospective longitudinal study is required to determine its sensitivity and responsiveness.

### 4.2 Strengths and limitations

The development and validation of a measure is an ongoing process, with data on psychometric properties being generated over time and not achievable in a single study. Although this study thoroughly evaluated the factor structure of the MRB-QoL Arabic version, its cross-sectional design limits the capability to assess tool's stability over time. This could be addressed in future longitudinal studies. Despite this limitation, using a clustered sample drawn from 4 different hospitals enhanced the findings' generalisability and suggested the tool's suitability across diverse healthcare settings in the UAE.

## 5 Conclusion

The findings of this study support the factor structure proposed in prior research, underscoring the validity and reliability of the Arabic MRB-QoL tool. This tool can be used to assess MRB from the patient's perspective and facilitate person-centred care in medication optimisation services across Arabic-speaking countries. Future work should explore the sensitivity, responsiveness, and clinical utility.

## CRediT authorship contribution statement

**Sundos Q. Al-Ebrahim:** Writing – original draft, Project administration, Methodology, Formal analysis, Data curation. **Khadija Hafidh:** Writing – review & editing, Methodology. **Mahir Jallo:** Project administration. **Mais M. Mauwfak:** Project administration. **Mohamed Nassef:** Project administration. **Hamzah Alzubaidi:** Supervision, Project administration. **Jeff Harrison:** Supervision, Methodology. **Timothy F. Chen:** Supervision, Methodology, Conceptualization. **Mohammed A. Mohammed:** Writing- review & editing. Supervision, Resources, Project administration, Methodology, Conceptualization.

## Declaration of competing interest

No conflict of interest.
